# Correction: A pragmatic pipeline for drug resistance and lineage identification in *Mycobacterium tuberculosis* using whole genome sequencing

**DOI:** 10.1371/journal.pgph.0005093

**Published:** 2025-08-21

**Authors:** Linzy Elton, Alp Aydin, Neil Stoker, Sylvia Rofael, Letícia Muraro Wildner, Jabar Babatunde Pacome Agbo Achimi Abdul, John Tembo, Muzamil Abdel Hamid, Mfoutou Mapanguy Claujens Chastel, Julio Ortiz Canseco, Ronan Doyle, Giovanni Satta, Justin O’Grady, Adam Witney, Francine Ntoumi, Alimuddin Zumla, Timothy D. McHugh

The Funding statement for this article is incorrect. The correct Funding statement is as follows: This publication was produced by CANTAM3 which is part of the EDCTP2 programme (grant number CSA2020NoE-3100) supported by the European Union. The views and opinions of authors expressed herein do not necessarily state or reflect those of EDCTP.
