# Supplementary material for: A pragmatic pipeline for drug resistance and lineage identification in Mycobacterium tuberculosis using whole genome sequencing
Source: PLOS Glob Public Health. 2025 Feb 10;5(2):e0004099. doi: 10.1371/journal.pgph.0004099 (PMC11809915; doi:10.1371/journal.pgph.0004099)
Supplement: S1 Text — (DOCX) [file pgph.0004099.s001.docx]

# **S2 Text1 – Full column CTAB protocol**

500 µL of liquid culture or a loopful of solid culture was resuspended in 500 µL PBS in a 2 mL MPBio tube, heat killed, washed twice with PBS and resuspended in 100 µL TE buffer (10mM Tris pH8 and 1mM EDTA). 2 x 4 mm glass beads were added and the samples were vortexed for 45 seconds. 10 µL lysozyme (10 mg/mL) was added and the samples then incubated at 37°C for 15 minutes at 800 RPM. The samples were then transferred into 1.5 mL Eppendorf DNA LoBind tubes and 10 µL proteinase K and 3 µL RNase A added, provided with the NEB Monarch Genomic Purification Kit (T3010S, New England Biolabs). This was vortexed, 200 µL CTAB buffer (2% CTAB, 40mM EDTA, 1.4M NaCl and 100mM Tris pH8.0) added and vortexed again. The samples were incubated at 56°C for 30 minutes at 1000 RPM, then 100 µL 5M NaCL added. The tubes were centrifuged at 17,000 x *g* for 5 minutes and 200 µL of the supernatant retained, avoiding the pellet.

The Monarch Genomic Purification Kit manufacturer instructions were then followed, with these alterations: 400 µL gDNA binding buffer was added to the supernatant and vortexed for 10 seconds. The lysate/binding buffer mix was transferred to a gDNA purification column, centrifuged at 1,000 x g for 3 minutes and then maximum speed for 1 minute to clear the membrane. 500 µL gDNA wash buffer was added and centrifuged for 1 minute at maximum speed and then repeated. The gDNA purification column was then placed in a DNase-free 2.5 mL microfuge tube and 50-100 µL preheated 60°C gDNA elution buffer added. The tubes were incubated at room temperature for 1 minute and then centrifuged at maximum speed for 1 minute to elute the gDNA. DNA was eluted into 50 µL molecular grade water.
