# Supplementary material for: A pragmatic pipeline for drug resistance and lineage identification in Mycobacterium tuberculosis using whole genome sequencing
Source: PLOS Glob Public Health. 2025 Feb 10;5(2):e0004099. doi: 10.1371/journal.pgph.0004099 (PMC11809915; doi:10.1371/journal.pgph.0004099)
Supplement: S1 Table — (DOCX) [file pgph.0004099.s002.docx]

# **S1 Table1– timings for methods tested**

|  | **Comparators** | | |
| --- | --- | --- | --- |
| **Culture length** | MGIT or 7H11  2 weeks | MGIT or 7H11  3 weeks | MGIT or 7H11  4 weeks |
| **DNA extraction (12 samples)** | Precipitation CTAB  1.5 days | Spin-column CTAB  1 day |  |
| **Library preparation (12 samples)** | RPB004 (PCR)  ~6 hours | RBK004 (native)  ~2 hours | RBK110.96 (native)  ~2 hours |
| **Basecalling algorithm (per barcode)** | Fast  15 minutes | HAC  205 minutes | SUP  >36 hours |
| **Analysis software** | TB-Profiler  Computing power/server speed dependent | Mykrobe  Computing power/server speed dependent |  |
