# Supplementary material for: A pragmatic pipeline for drug resistance and lineage identification in Mycobacterium tuberculosis using whole genome sequencing
Source: PLOS Glob Public Health. 2025 Feb 10;5(2):e0004099. doi: 10.1371/journal.pgph.0004099 (PMC11809915; doi:10.1371/journal.pgph.0004099)
Supplement: S2 Table — (DOCX) [file pgph.0004099.s003.docx]

# **S1 Table2 - Computer specifications for the computer used in this project**

| **Component Required** | **Specification** |
| --- | --- |
| Operating system | Windows 10 |
| Memory/RAM | 32 GB RAM |
| CPU | Intel(R) Core(TM) i7-10700 CPU @ 2.90GHz 2.90 GHz |
| Storage | 1 TB internal SSD |
| Ports | USB 3.0 |
