# Supplementary material for: A pragmatic pipeline for drug resistance and lineage identification in Mycobacterium tuberculosis using whole genome sequencing
Source: PLOS Glob Public Health. 2025 Feb 10;5(2):e0004099. doi: 10.1371/journal.pgph.0004099 (PMC11809915; doi:10.1371/journal.pgph.0004099)
Supplement: S3 Table — (DOCX) [file pgph.0004099.s004.docx]

# **S1 Table3 - List of uploaded fastq files**

| **Isolate name** | **Sequencing platform** | **Sample Accession number** | **Experiment Accession number** |
| --- | --- | --- | --- |
| TB_sensitive_1 | Oxford Nanopore Technologies | ERS16613404 | ERX11656854 |
| TB_sensitive_2 | Oxford Nanopore Technologies | ERS16613405 | ERX11656855 |
| TB_sensitive_3 | Oxford Nanopore Technologies | ERS16613406 | ERX11656856 |
| TB_sensitive_4 | Oxford Nanopore Technologies | ERS16613407 | ERX11656857 |
| TB_sensitive_5 | Oxford Nanopore Technologies | ERS16613408 | ERX11656858 |
| TB_sensitive_6 | Oxford Nanopore Technologies | ERS16613409 | ERX11656859 |
| TB_sensitive_7 | Oxford Nanopore Technologies | ERS16613410 | ERX11656860 |
| TB_sensitive_8 | Oxford Nanopore Technologies | ERS16613411 | ERX11656861 |
| TB_isoniazid_mono_1 | Oxford Nanopore Technologies | ERS16613412 | ERX11656845 |
| TB_isoniazid_mono_2 | Oxford Nanopore Technologies | ERS16613413 | ERX11656846 |
| TB_isoniazid_mono_3 | Oxford Nanopore Technologies | ERS16613415 | ERX11656847 |
| TB_isoniazid_mono_4 | Oxford Nanopore Technologies | ERS16613416 | ERX11656848 |
| TB_isoniazid_mono_5 | Oxford Nanopore Technologies | ERS16613417 | ERX11656849 |
| TB_MDR_1 | Oxford Nanopore Technologies | ERS16613418 | ERX11656850 |
| TB_MDR_2 | Oxford Nanopore Technologies | ERS16613419 | ERX11656851 |
| TB_MDR_3 | Oxford Nanopore Technologies | ERS16613420 | ERX11656852 |
| TB_MDR_4 | Oxford Nanopore Technologies | ERS16613421 | ERX11656853 |
| TB_sensitive_1_1 | Illumina | ERS16690010 | ERX11656882 |
| TB_sensitive_1_2 | Illumina | ERS16690011 | ERX11656883 |
| TB_sensitive_2_1 | Illumina | ERS16690012 | ERX11656884 |
| TB_sensitive_2_2 | Illumina | ERS16690013 | ERX11656885 |
| TB_sensitive_3_1 | Illumina | ERS16690014 | ERX11656886 |
| TB_sensitive_3_2 | Illumina | ERS16690015 | ERX11656887 |
| TB_sensitive_4_1 | Illumina | ERS16690016 | ERX11656888 |
| TB_sensitive_4_2 | Illumina | ERS16690017 | ERX11656889 |
| TB_sensitive_5_1 | Illumina | ERS16690018 | ERX11656890 |
| TB_sensitive_5_2 | Illumina | ERS16690019 | ERX11656891 |
| TB_sensitive_6_1 | Illumina | ERS16690020 | ERX11656892 |
| TB_sensitive_6_2 | Illumina | ERS16690021 | ERX11656893 |
| TB_sensitive_7_1 | Illumina | ERS16690022 | ERX11656894 |
| TB_sensitive_7_2 | Illumina | ERS16690023 | ERX11656895 |
| TB_sensitive_8_1 | Illumina | ERS16690024 | ERX11656896 |
| TB_sensitive_8_2 | Illumina | ERS16690025 | ERX11656897 |
| TB_isoniazid_mono_1_1 | Illumina | ERS16690026 | ERX11656864 |
| TB_isoniazid_mono_1_2 | Illumina | ERS16690027 | ERX11656865 |
| TB_isoniazid_mono_2_1 | Illumina | ERS16690028 | ERX11656866 |
| TB_isoniazid_mono_2_2 | Illumina | ERS16690029 | ERX11656867 |
| TB_isoniazid_mono_3_1 | Illumina | ERS16690030 | ERX11656868 |
| TB_isoniazid_mono_3_2 | Illumina | ERS16690031 | ERX11656869 |
| TB_isoniazid_mono_4_1 | Illumina | ERS16690032 | ERX11656870 |
| TB_isoniazid_mono_4_2 | Illumina | ERS16690033 | ERX11656871 |
| TB_isoniazid_mono_5_1 | Illumina | ERS16690034 | ERX11656872 |
| TB_isoniazid_mono_5_2 | Illumina | ERS16690035 | ERX11656873 |
| TB_MDR_1_1 | Illumina | ERS16690036 | ERX11656874 |
| TB_MDR_1_2 | Illumina | ERS16690037 | ERX11656875 |
| TB_MDR_2_1 | Illumina | ERS16690038 | ERX11656876 |
| TB_MDR_2_2 | Illumina | ERS16690039 | ERX11656877 |
| TB_MDR_3_1 | Illumina | ERS16690040 | ERX11656878 |
| TB_MDR_3_2 | Illumina | ERS16690041 | ERX11656879 |
| TB_MDR_4_1 | Illumina | ERS16690042 | ERX11656880 |
| TB_MDR_4_2 | Illumina | ERS16690043 | ERX11656881 |
