# Supplementary material for: A pragmatic pipeline for drug resistance and lineage identification in Mycobacterium tuberculosis using whole genome sequencing
Source: PLOS Glob Public Health. 2025 Feb 10;5(2):e0004099. doi: 10.1371/journal.pgph.0004099 (PMC11809915; doi:10.1371/journal.pgph.0004099)
Supplement: S4 Table — (DOCX) [file pgph.0004099.s005.docx]

# **S1 Table4 - Full basecalling comparison table**

|  | Sample | Basecaller | **Lineage** | **Lineage name** | **Rifampicin** | **Isoniazid** | **Ethambutol** | **Streptomycin** | **Moxifloxacin** | **Ofloxaxin** | **Amikacin** | **Capreomycin** | **Kanamycin** | **Pyrazinamide** | **Ethionamide** | **Ciprofloxacin** | **Fluoroquinolones** | **PAS** | **Cycloserine** | **Delaminid** |
| --- | --- | --- | --- | --- | --- | --- | --- | --- | --- | --- | --- | --- | --- | --- | --- | --- | --- | --- | --- | --- |
| Sensitive | S1 | F | 2.2.1 | Beijing | Se | Se | Se | Se | Se | Se | Se | Se | Se | Se | Se | Se | Se | Se | Se | Se |
|  |  | H | 2.2.1 | Beijing | Se | Se | Se | Se | Se | Se | Se | Se | Se | Se | Se | Se | Se | Se | Se | Se |
|  |  | S | 2.2.1 | Beijing | Se | Se | Se | Se | Se | Se | Se | Se | Se | Se | Se | Se | Se | Se | Se | Se |
|  |  |  |  |  |  |  |  |  |  |  |  |  |  |  |  |  |  |  |  |  |
|  | S2 | F | 4.1.2.1 | None | Se | Se | Se | rpsL p.Lys43Arg | Se | Se | Se | Se | Se | Se | Se | Se | Se | Se | Se | Se |
|  |  | H | 4.1.2.1 | T1 | Se | Se | Se | rpsL p.Lys43Arg | Se | Se | Se | Se | Se | Se | Se | Se | Se | Se | Se | Se |
|  |  | S | 4.1.2.1 | T1 | Se | Se | Se | rpsL p.Lys43Arg | Se | Se | Se | Se | Se | Se | Se | Se | Se | Se | Se | Se |
|  |  |  |  |  |  |  |  |  |  |  |  |  |  |  |  |  |  |  |  |  |
|  | S3 | F | 4.1.1.1 | X2 | Se | Se | Se | Se | Se | Se | Se | Se | Se | Se | Se | Se | Se | Se | Se | Se |
|  |  | H | 4.1.1.1 | X2 | Se | Se | Se | Se | Se | Se | Se | Se | Se | Se | Se | Se | Se | Se | Se | Se |
|  |  | S | 4.1.1.1 | X2 | Se | Se | Se | Se | Se | Se | Se | Se | Se | Se | Se | Se | Se | Se | Se | Se |
|  |  |  |  |  |  |  |  |  |  |  |  |  |  |  |  |  |  |  |  |  |
|  | S4 | F | 3 | CAS | Se | Se | Se | Se | Se | Se | Se | Se | Se | Se | Se | Se | Se | Se | Se | Se |
|  |  | H | 4.6 | Manu2 | Se | Se | Se | Se | Se | Se | Se | Se | Se | Se | Se | Se | Se | Se | Se | Se |
|  |  | S | 4.6 | Manu2 | Se | Se | Se | Se | Se | Se | Se | Se | Se | Se | Se | Se | Se | Se | Se | Se |
|  |  |  |  |  |  |  |  |  |  |  |  |  |  |  |  |  |  |  |  |  |
|  | S5 | F | 4.3.4 | None | Se | Se | Se | Se | Se | Se | Se | Se | Se | Se | Se | Se | Se | Se | Se | Se |
|  |  | H | 4.3.4.2 | LAM9 | Se | Se | Se | Se | Se | Se | Se | Se | Se | Se | Se | Se | Se | Se | Se | Se |
|  |  | S | 4.3.4.2 | LAM9 | Se | Se | Se | Se | Se | Se | Se | Se | Se | Se | Se | Se | Se | Se | Se | Se |
|  |  |  |  |  |  |  |  |  |  |  |  |  |  |  |  |  |  |  |  |  |
|  | S6 | F | 2.2.1 | Beijing | Se | Se | Se | Se | Se | Se | Se | Se | Se | Se | Se | Se | Se | Se | Se | Se |
|  |  | H | 2.2.1 | Beijing | Se | Se | Se | Se | Se | Se | Se | Se | Se | Se | Se | Se | Se | Se | Se | Se |
|  |  | S | 2.2.1 | Beijing | Se | Se | Se | Se | Se | Se | Se | Se | Se | Se | Se | Se | Se | Se | Se | Se |
|  |  |  |  |  |  |  |  |  |  |  |  |  |  |  |  |  |  |  |  |  |
|  | S7 | F | 4.1.3 | None | Se | Se | Se | Se | Se | Se | Se | Se | Se | Se | Se | Se | Se | Se | Se | Se |
|  |  | H | 4.1.3 | T1 | Se | Se | Se | Se | Se | Se | Se | Se | Se | Se | Se | Se | Se | Se | Se | Se |
|  |  | S | 4.1.3 | T1 | Se | Se | Se | Se | Se | Se | Se | Se | Se | Se | Se | Se | Se | Se | Se | Se |
|  |  |  |  |  |  |  |  |  |  |  |  |  |  |  |  |  |  |  |  |  |
|  | S8 | F | 1.2.1.2.1 | EAI2-nonthaburi | Se | Se | Se | Se | Se | Se | Se | Se | Se | Se | Se | Se | Se | Se | Se | Se |
|  |  | H | 1.2.1.2.1 | EAI2-nonthaburi | Se | Se | Se | Se | Se | Se | Se | Se | Se | Se | Se | Se | Se | Se | Se | Se |
|  |  | S | 1.2.1.2.1 | EAI2-nonthaburi | Se | Se | Se | Se | Se | Se | Se | Se | Se | Se | Se | Se | Se | Se | Se | Se |
|  |  |  |  |  |  |  |  |  |  |  |  |  |  |  |  |  |  |  |  |  |
| Isoniazid mono-resistant | I1 | F | 4.6.2.2 | None | Se | fabG1 c.-15C>T | Se | Se | Se | Se | Se | Se | Se | Se | fabG1 c.-15C>T | Se | Se | Se | Se | Se |
|  |  | H | 4.6.2.2 | Cameroon | Se | fabG1 c.-15C>T | Se | Se | Se | Se | Se | Se | Se | Se | fabG1 c.-15C>T | Se | Se | Se | Se | Se |
|  |  | S | 4.6.2.3 | Cameroon | Se | fabG1 c.-15C>T | Se | Se | Se | Se | Se | Se | Se | Se | fabG1 c.-15C>T | Se | Se | Se | Se | Se |
|  |  |  |  |  |  |  |  |  |  |  |  |  |  |  |  |  |  |  |  |  |
|  | I2 | F | 4.6.1.2 | None | Se | fabG1 c.-15C>T, inhA p.Ile194Thr | Se | Se | Se | Se | Se | Se | Se | Se | fabG1 c.-15C>T, inhA p.Ile194Thr | Se | Se | Se | Se | Se |
|  |  | H | 4.6.1.2 | X1 | Se | fabG1 c.-15C>T, inhA p.Ile194Thr | Se | gid c.102de1G | Se | Se | Se | Se | Se | Se | fabG1 c.-15C>T, inhA p.Ile194Thr | Se | Se | Se | Se | Se |
|  |  | S | 4.6.1.2 | X1 | Se | fabG1 c.-15C>T, inhA p.Ile194Thr | Se | gid c.102de1G | Se | Se | Se | Se | Se | Se | fabG1 c.-15C>T, inhA p.Ile194Thr | Se | Se | Se | Se | Se |
|  |  |  |  |  |  |  |  |  |  |  |  |  |  |  |  |  |  |  |  |  |
|  | I3 | F | 4.6.2.2 | Cameroon | Se | fabG1 c.-15C>T | Se | Se | Se | Se | Se | Se | Se | Se | fabG1 c.-15C>T | Se | Se | Se | Se | Se |
|  |  | H | 4.6.2.2 | Cameroon | Se | fabG1 c.-15C>T | Se | Se | Se | Se | Se | Se | Se | Se | fabG1 c.-15C>T | Se | Se | Se | Se | Se |
|  |  | S | 4.6.2.2 | Cameroon | Se | fabG1 c.-15C>T | Se | Se | Se | Se | Se | Se | Se | Se | fabG1 c.-15C>T | Se | Se | Se | Se | Se |
|  |  |  |  |  |  |  |  |  |  |  |  |  |  |  |  |  |  |  |  |  |
|  | I4 | F | 4.6.2.2 | Cameroon | Se | fabG1 c.-15C>T | Se | Se | Se | Se | Se | Se | Se | Se | fabG1 c.-15C>T | Se | Se | Se | Se | Se |
|  |  | H | 4.6.2.2 | Cameroon | Se | fabG1 c.-15C>T | Se | Se | Se | Se | Se | Se | Se | Se | fabG1 c.-15C>T | Se | Se | Se | Se | Se |
|  |  | S | 4.6.2.2 | Cameroon | Se | fabG1 c.-15C>T | Se | Se | Se | Se | Se | Se | Se | Se | fabG1 c.-15C>T | Se | Se | Se | Se | Se |
|  |  |  |  |  |  |  |  |  |  |  |  |  |  |  |  |  |  |  |  |  |
|  | I5 | Fast | 4.6.2.2 | Cameroon | Se | fabG1 c.-15C>T | Se | Se | Se | Se | Se | Se | Se | Se | fabG1 c.-15C>T | Se | Se | Se | Se | Se |
|  |  | HAC | 4.6.2.2 | Cameroon | Se | fabG1 c.-15C>T | Se | Se | Se | Se | Se | Se | Se | Se | fabG1 c.-15C>T | Se | Se | Se | Se | Se |
|  |  | SUP | 4.6.2.2 | Cameroon | Se | fabG1 c.-15C>T | Se | Se | Se | Se | Se | Se | Se | Se | fabG1 c.-15C>T | Se | Se | Se | Se | Se |
|  |  |  |  |  |  |  |  |  |  |  |  |  |  |  |  |  |  |  |  |  |
| MDR | M1 | F | 4.1.2.1 | None | rpoB p.Ser450Leu | katG p.Ser315Thr | embB p.Met306Ile | rpsL p.Lys43Arg | Se | Se | Se | Se | Se | pncA p.Leu85Pro | Se | Se | Se | Se | Se | Se |
|  |  | H | 4.1.2.1 | H1 | rpoB p.Ser450Leu | katG p.Ser315Thr | embB p.Met306Ile | rpsL p.Lys43Arg | Se | Se | Se | Se | Se | pncA p.Leu85Pro | Se | Se | Se | Se | Se | Se |
|  |  | S | 4.1.2.1 | H1 | rpoB p.Ser450Leu | katG p.Ser315Thr | embB p.Met306Ile | rpsL p.Lys43Arg | Se | Se | Se | Se | Se | pncA p.Leu85Pro | Se | Se | Se | Se | Se | Se |
|  |  |  |  |  |  |  |  |  |  |  |  |  |  |  |  |  |  |  |  |  |
|  | M2 | F | 2.2.1 | Beijing | rpoB p.Ser450Leu, rpoB p.Glu761Asp | katG p.Ser315Thr | embB p.Asp354Ala | Se | Se | Se | Se | Se | eis c.-37G>T | Se | ethA c.-7T>C | Se | Se | Se | Se | Se |
|  |  | H | 2.2.1 | Beijing | rpoB p.Ser450Leu, rpoB p.Glu761Asp | katG p.Ser315Thr | embB p.Asp354Ala | Se | Se | Se | Se | Se | eis c.-37G>T | Se | ethA c.-7T>C | Se | Se | Se | Se | Se |
|  |  | S | 2.2.1 | Beijing | rpoB p.Ser450Leu, rpoB p.Glu761Asp | katG p.Ser315Thr | embB p.Asp354Ala | Se | Se | Se | Se | Se | eis c.-37G>T | Se | ethA c.-7T>C | Se | Se | Se | Se | Se |
|  |  |  |  |  |  |  |  |  |  |  |  |  |  |  |  |  |  |  |  |  |
|  | M3 | F | 2.2.1 | Beijing | rpoB p.Ser450Leu, rpoB p.Glu761Asp | katG p.Ser315Thr | embB p.Asp354Ala | Se | Se | Se | Se | Se | eis c.-37G>T | Se | ethA c.-7T>C | Se | Se | Se | Se | Se |
|  |  | H | 2.2.1 | Beijing | rpoB p.Ser450Leu, rpoB p.Glu761Asp | katG p.Ser315Thr | embB p.Asp354Ala | Se | Se | Se | Se | Se | eis c.-37G>T | Se | ethA c.-7T>C | Se | Se | Se | Se | Se |
|  |  | S | 2.2.1 | Beijing | rpoB p.Ser450Leu, rpoB p.Glu761Asp | katG p.Ser315Thr | embB p.Asp354Ala | Se | Se | Se | Se | Se | eis c.-37G>T | Se | ethA c.-7T>C | Se | Se | Se | Se | Se |
|  |  |  |  |  |  |  |  |  |  |  |  |  |  |  |  |  |  |  |  |  |
|  | M4 | F | 4.2.1 | Euro-American (TUR) | rpoB p.Ser450Leu, rpoC p.Asp485Asn | inhA c.-154G>A, katG p.Ser315Thr | Se | rpsL p.Lys88Arg | Se | Se | Se | Se | Se | Se | inhA c.-154G>A | Se | Se | Se | Se | Se |
|  |  | H | 4.2.1 | Ural-1 | rpoB p.Ser450Leu, rpoC p.Asp485Asn | inhA c.-154G>A, katG p.Ser315Thr | Se | rpsL p.Lys88Arg | Se | Se | Se | Se | Se | Se | inhA c.-154G>A | Se | Se | Se | Se | Se |
|  |  | S | 4.2.1 | Ural-1 | rpoB p.Ser450Leu, rpoC p.Asp485Asn | inhA c.-154G>A, katG p.Ser315Thr | Se | rpsL p.Lys88Arg | Se | Se | Se | Se | Se | Se | inhA c.-154G>A | Se | Se | Se | Se | Se |

F=fast basecalling, H= high accuracy basecalling, S= super high accuracy basecalling, Se=sensitive
