# Supplementary material for: A pragmatic pipeline for drug resistance and lineage identification in Mycobacterium tuberculosis using whole genome sequencing
Source: PLOS Glob Public Health. 2025 Feb 10;5(2):e0004099. doi: 10.1371/journal.pgph.0004099 (PMC11809915; doi:10.1371/journal.pgph.0004099)
Supplement: S5 Table — (DOCX) [file pgph.0004099.s006.docx]

# **S1 Table5 - Full tuberculosis data analysis software comparison table**

|  | Sample | Software | **Lineage** | **Lineage name** | **Rifampicin** | **Isoniazid** | **Ethambutol** | **Streptomycin** | **Moxifloxacin** | **Ofloxaxin** | **Amikacin** | **Capreomycin** | **Kanamycin** | **Pyrazinamide** | **Ethionamide** | **Ciprofloxacin** | **Fluoroquinolones** | **PAS** | **Cycloserine** | **Delaminid** |
| --- | --- | --- | --- | --- | --- | --- | --- | --- | --- | --- | --- | --- | --- | --- | --- | --- | --- | --- | --- | --- |
| Sensitive | S1 | T | 2.2.1 | Beijing | S | S | S | S | S | S | S | S | S | S | S | S | S | S | S | S |
|  |  | M | 2.2.10 | n/a | S | S | S | S | S | S | S | S | S | S | NT | S | NT | NT | NT | NT |
|  |  |  |  |  |  |  |  |  |  |  |  |  |  |  |  |  |  |  |  |  |
|  | S2 | T | 4.1.2.1 | T1 | S | S | S | rpsL p.Lys43Arg | S | S | S | S | S | S | S | S | S | S | S | S |
|  |  | M | 4.1.2.1 | n/a | S | S | S | rpsL p.Lys43Arg | S | S | S | S | S | S | NT | S | NT | NT | NT | NT |
|  |  |  |  |  |  |  |  |  |  |  |  |  |  |  |  |  |  |  |  |  |
|  | S3 | T | 4.1.1.1 | X2 | S | S | S | S | S | S | S | S | S | S | S | S | S | S | S | S |
|  |  | M | 4.1.1.1 | n/a | S | S | S | S | S | S | S | S | S | S | NT | S | NT | NT | NT | NT |
|  |  |  |  |  |  |  |  |  |  |  |  |  |  |  |  |  |  |  |  |  |
|  | S4 | T | 4.6 | Manu2 | S | S | S | S | S | S | S | S | S | S | S | S | S | S | S | S |
|  |  | M | 3 and 4.6.2.2 | n/a | S | C in gene fabG1 | S | S | S | S | S | S | S | S | NT | S | NT | NT | NT | NT |
|  |  |  |  |  |  |  |  |  |  |  |  |  |  |  |  |  |  |  |  |  |
|  | S5 | T | 4.3.4 | LAM9 | S | S | S | S | S | S | S | S | S | S | S | S | S | S | S | S |
|  |  | M | 4.3.4.2 | n/a | S | S | S | S | S | S | S | S | S | S | NT | S | NT | NT | NT | NT |
|  |  |  |  |  |  |  |  |  |  |  |  |  |  |  |  |  |  |  |  |  |
|  | S6 | T | 2.2.1 | Beijing | S | S | S | S | S | S | S | S | S | S | S | S | S | S | S | S |
|  |  | M | 2.2 | n/a | S | S | S | S | S | S | S | S | S | S | NT | S | NT | NT | NT | NT |
|  |  |  |  |  |  |  |  |  |  |  |  |  |  |  |  |  |  |  |  |  |
|  | S7 | T | 4.1.3 | T1 | S | S | S | S | S | S | S | S | S | S | S | S | S | S | S | S |
|  |  | M | 4.1.3 | n/a | S | S | S | S | S | S | S | S | S | S | NT | S | NT | NT | NT | NT |
|  |  |  |  |  |  |  |  |  |  |  |  |  |  |  |  |  |  |  |  |  |
|  | S8 | T | 1.2.1.2.1 | EAI2-nonthaburi | S | S | S | S | S | S | S | S | S | S | S | S | S | S | S | S |
|  |  | M | 1.2.1 | n/a | S | S | S | S | S | S | S | S | S | S | S | S | S | S | S | S |
|  |  |  |  |  |  |  |  |  |  |  |  |  |  |  |  |  |  |  |  |  |
| Isoniazid mono resistance | I1 | T | 4.6.2.2 | Cameroon | S | fabG1 c.-15C>T | S | S | S | S | S | S | S | S | fabG1 c.-15C>T | S | S | S | S | S |
|  |  | M | 4.6.2.2 | n/a | S | C in gene fabG1 | S | S | S | S | S | S | S | S | NT | S | NT | NT | NT | NT |
|  |  |  |  |  |  |  |  |  |  |  |  |  |  |  |  |  |  |  |  |  |
|  | I2 | T | 4.6.1.2 | X1 | S | fabG1 c.-15C>T, inhA p.Ile194Thr | S | S | S | S | S | S | S | S | fabG1 c.-15C>T, inhA p.Ile194Thr | S | S | S | S | S |
|  |  | M | 4.6.1.2 | n/a | S | inhA p.Ile194Thr  C in gene fabG1 | S | S | S | S | S | S | S | S | NT | S | NT | NT | NT | NT |
|  |  |  |  |  |  |  |  |  |  |  |  |  |  |  |  |  |  |  |  |  |
|  | I3 | T | 4.6.2.2 | Cameroon | S | fabG1 c.-15C>T | S | S | S | S | S | S | S | S | fabG1 c.-15C>T | S | S | S | S | S |
|  |  | M | 4.6.2.2 | n/a | S | C in gene fabG1 | S | S | S | S | S | S | S | S | NT | S | NT | NT | NT | NT |
|  |  |  |  |  |  |  |  |  |  |  |  |  |  |  |  |  |  |  |  |  |
|  | I4 | T | 4.6.2.2 | Cameroon | S | fabG1 c.-15C>T | S | S | S | S | S | S | S | S | fabG1 c.-15C>T | S | S | S | S | S |
|  |  | M | 4.6.2.2 | n/a | S | C in gene fabG1 | S | S | S | S | S | S | S | S | NT | S | NT | NT | NT | NT |
|  |  |  |  |  |  |  |  |  |  |  |  |  |  |  |  |  |  |  |  |  |
|  | I5 | T | 4.6.2.2 | Cameroon | S | fabG1 c.-15C>T | S | S | S | S | S | S | S | S | fabG1 c.-15C>T | S | S | S | S | S |
|  |  | M | 4.6.2.2 | n/a | S | C in gene fabG1 | S | S | S | S | S | S | S | S | NT | S | NT | NT | NT | NT |
|  |  |  |  |  |  |  |  |  |  |  |  |  |  |  |  |  |  |  |  |  |
| MDR | M1 | T | 4.1.2.1 | H1 | rpoB p.Ser450Leu | katG p.Ser315Thr | M306I in gene embB | rpsL p.Lys43Arg | S | S | S | S | S | pncA p.Leu85Pro | S | S | S | S | S | S |
|  |  | M | 4.1.2.1 | n/a | rpoB p.Ser450Leu | katG p.Ser315Thr | M306I in gene embB | rpsL p.Lys43Arg | S | S | S | S | S | pncA p.Leu85Pro | NT | S | NT | NT | NT | NT |
|  |  |  |  |  |  |  |  |  |  |  |  |  |  |  |  |  |  |  |  |  |
|  | M2 | T | 2.2.1 | Beijing | rpoB p.Ser450Leu, rpoB p.Glu761Asp | katG p.Ser315Thr | embB p.Asp354Ala | S | S | S | S | S | eis c.-37G>T | S | ethA c.-7T>C | S | S | S | S | S |
|  |  | M | 2.2.10 | n/a | rpoB p.Ser450Leu, rpoB p.Glu761Asp | katG p.Ser315Thr | embB p.Asp354Ala | S | S | S | S | S | S | S | NT | S | NT | NT | NT | NT |
|  |  |  |  |  |  |  |  |  |  |  |  |  |  |  |  |  |  |  |  |  |
|  | M3 | T | 2.2.1 | Beijing | rpoB p.Ser450Leu, rpoB p.Glu761Asp | katG p.Ser315Thr | embB p.Asp354Ala | S | S | S | S | S | eis c.-37G>T | S | ethA c.-7T>C | S | S | S | S | S |
|  |  | M | 2.2.10 | n/a | S450L in gene rpoB | katG p.Ser315Thr | embB p.Asp354Ala | S | S | S | S | S | S | S | NT | S | NT | NT | NT | NT |
|  |  |  |  |  |  |  |  |  |  |  |  |  |  |  |  |  |  |  |  |  |
|  | M4 | T | 4.2.1 | Ural-1 | rpoB p.Ser450Leu, rpoC p.Asp485Asn | inhA c.-154G>A, katG p.Ser315Thr | S | rpsL p.Lys88Arg | S | S | S | S | S | S | inhA c.-154G>A | S | S | S | S | S |
|  |  | M | 4.2.1 | n/a | S | katG p.Ser315Thr | S | S | S | S | S | S | S | S | NT | S | NT | NT | NT | NT |

T= TB-Profiler, M= Mykrobe, S= sensitive, NT = not tested
