# Supplementary material for: A pragmatic pipeline for drug resistance and lineage identification in Mycobacterium tuberculosis using whole genome sequencing
Source: PLOS Glob Public Health. 2025 Feb 10;5(2):e0004099. doi: 10.1371/journal.pgph.0004099 (PMC11809915; doi:10.1371/journal.pgph.0004099)
Supplement: S6 Table — (DOCX) [file pgph.0004099.s007.docx]

# **S1 Table6 - Full ONT vs Illumina concordance table**

|  |  |  | **Lineage** | **Lineage name** | **Rifampicin** | **Isoniazid** | **Ethambutol** | **Streptomycin** | **Moxifloxacin** | **Ofloxaxin** | **Amikacin** | **Capreomycin** | **Kanamycin** | **Pyrazinamide** | **Ethionamide** | **Ciprofloxacin** | **Fluoroquinolones** | **PAS** | **Cycloserine** | **Delaminid** |
| --- | --- | --- | --- | --- | --- | --- | --- | --- | --- | --- | --- | --- | --- | --- | --- | --- | --- | --- | --- | --- |
| **Sensitive** | S1 | P | n/a | n/a | S | S | S | S | NT | NT | NT | NT | NT | NT | NT | NT | NT | NT | NT | NT |
|  |  | O | 2.2.1 | Beijing | S | S | S | S | S | S | S | S | S | S | S | S | S | S | S | S |
|  |  | I | 2.2.1 | Beijing | S | S | S | S | S | S | S | S | S | S | S | S | S | S | S | S |
|  |  |  |  |  |  |  |  |  |  |  |  |  |  |  |  |  |  |  |  |  |
|  | S2 | P | n/a | n/a | S | S | S | S | NT | NT | NT | NT | NT | NT | NT | NT | NT | NT | NT | NT |
|  |  | O | 4.1.2.1 | T1 | S | S | S | rpsL p.Lys43Arg | S | S | S | S | S | S | S | S | S | S | S | S |
|  |  | I | 4.1.2.1 | T1 | S | S | S | rpsL p.Lys43Arg | S | S | S | S | S | S | S | S | S | S | S | S |
|  |  |  |  |  |  |  |  |  |  |  |  |  |  |  |  |  |  |  |  |  |
|  | S3 | P | n/a | n/a | S | S | S | S | NT | NT | NT | NT | NT | NT | NT | NT | NT | NT | NT | NT |
|  |  | O | 4.1.1.1 | X2 | S | S | S | S | S | S | S | S | S | S | S | S | S | S | S | S |
|  |  | I | 4.1.1.1 | X2 | S | S | S | S | S | S | S | S | S | S | S | S | S | S | S | S |
|  |  |  |  |  |  |  |  |  |  |  |  |  |  |  |  |  |  |  |  |  |
|  | S4 | P | n/a | n/a | S | S | S | S | NT | NT | NT | NT | NT | NT | NT | NT | NT | NT | NT | NT |
|  |  | O | 3 and 4.6 | CAS, Manu2 | S | S | S | S | S | S | S | S | S | S | S | S | S | S | S | S |
|  |  | I | 3 | CAS1-Delhi | S | S | S | S | S | S | S | S | S | S | S | S | S | S | S | S |
|  |  |  |  |  |  |  |  |  |  |  |  |  |  |  |  |  |  |  |  |  |
|  | S5 | P | n/a | n/a | S | S | S | S | NT | NT | NT | NT | NT | NT | NT | NT | NT | NT | NT | NT |
|  |  | O | 4.3.4 | LAM9 | S | S | S | S | S | S | S | S | S | S | S | S | S | S | S | S |
|  |  | I | 4.3.4.2 | LAM9 | S | S | S | S | S | S | S | S | S | S | S | S | S | S | S | S |
|  |  |  |  |  |  |  |  |  |  |  |  |  |  |  |  |  |  |  |  |  |
|  | S6 | P | n/a | n/a | S | S | S | S | NT | NT | NT | NT | NT | NT | NT | NT | NT | NT | NT | NT |
|  |  | O | 2.2.1 | Beijing | S | S | S | S | S | S | S | S | S | S | S | S | S | S | S | S |
|  |  | I | 2.2.1 | Beijing | S | S | S | S | S | S | S | S | S | S | S | S | S | S | S | S |
|  |  |  |  |  |  |  |  |  |  |  |  |  |  |  |  |  |  |  |  |  |
|  | S7 | P | n/a | n/a | S | S | S | S | NT | NT | NT | NT | NT | NT | NT | NT | NT | NT | NT | NT |
|  |  | O | 4.1.3 | T1 | S | S | S | S | S | S | S | S | S | S | S | S | S | S | S | S |
|  |  | I | 4.1.3 | T1 | S | S | S | S | S | S | S | S | S | S | S | S | S | S | S | S |
|  |  |  |  |  |  |  |  |  |  |  |  |  |  |  |  |  |  |  |  |  |
|  | S8 | P | n/a | n/a | S | S | S | S | NT | NT | NT | NT | NT | NT | NT | NT | NT | NT | NT | NT |
|  |  | O | 1.2.1.2.1 | EAI2-nonthaburi | S | S | S | S | S | S | S | S | S | S | S | S | S | S | S | S |
|  |  | I | 1.2.1.2.1 | EAI2-nonthaburi | S | S | S | S | S | S | S | S | S | S | S | S | S | S | S | S |
|  |  |  |  |  |  |  |  |  |  |  |  |  |  |  |  |  |  |  |  |  |
| **Isoniazid mono resistant** | I1 | P | n/a | n/a | S | R | S | S | NT | NT | NT | NT | NT | NT | NT | NT | NT | NT | NT | NT |
|  |  | O | 4.6.2.2 | Cameroon | S | fabG1 c.-15C>T | S | S | S | S | S | S | S | S | fabG1 c.-15C>T | S | S | S | S | S |
|  |  | I | 4.6.2.2 | Cameroon | S | fabG1 c.-15C>T | S | S | S | S | S | S | S | S | fabG1 c.-15C>T | S | S | S | S | S |
|  |  |  |  |  |  |  |  |  |  |  |  |  |  |  |  |  |  |  |  |  |
|  | I2 | P | n/a | n/a | S | R | S | S | NT | NT | NT | NT | NT | NT | NT | NT | NT | NT | NT | NT |
|  |  | O | 4.6.1.2 | X1 | S | fabG1 c.-15C>T, inhA p.Ile194Thr | S | gid c.102delG | S | S | S | S | S | S | fabG1 c.-15C>T, inhA p.Ile194Thr | S | S | S | S | S |
|  |  | I | 4.6.1.2 | X1 | S | fabG1 c.-15C>T, inhA p.Ile194Thr | S | gid c.102delG | S | S | S | S | S | S | fabG1 c.-15C>T, inhA p.Ile194Thr | S | S | S | S | S |
|  |  |  |  |  |  |  |  |  |  |  |  |  |  |  |  |  |  |  |  |  |
|  | I3 | P | n/a | n/a | S | R | S | S | NT | NT | NT | NT | NT | NT | NT | NT | NT | NT | NT | NT |
|  |  | O | 4.6.2.2 | Cameroon | S | fabG1 c.-15C>T | S | S | S | S | S | S | S | S | fabG1 c.-15C>T | S | S | S | S | S |
|  |  | I | 4.6.2.2 | Cameroon | S | fabG1 c.-15C>T | S | S | S | S | S | S | S | S | fabG1 c.-15C>T | S | S | S | S | S |
|  |  |  |  |  |  |  |  |  |  |  |  |  |  |  |  |  |  |  |  |  |
|  | I4 | P | n/a | n/a | S | R | S | S | NT | NT | NT | NT | NT | NT | NT | NT | NT | NT | NT | NT |
|  |  | O | 4.6.2.2 | Cameroon | S | fabG1 c.-15C>T | S | S | S | S | S | S | S | S | fabG1 c.-15C>T | S | S | S | S | S |
|  |  | I | 4.6.2.2 | Cameroon | S | fabG1 c.-15C>T | S | S | S | S | S | S | S | S | fabG1 c.-15C>T | S | S | S | S | S |
|  |  |  |  |  |  |  |  |  |  |  |  |  |  |  |  |  |  |  |  |  |
|  | I5 | P | n/a | n/a | S | R | S | S | NT | NT | NT | NT | NT | NT | NT | NT | NT | NT | NT | NT |
|  |  | O | 4.6.2.2 | Cameroon | S | fabG1 c.-15C>T | S | S | S | S | S | S | S | S | fabG1 c.-15C>T | S | S | S | S | S |
|  |  | I | 4.6.2.2 | Cameroon | S | fabG1 c.-15C>T | S | S | S | S | S | S | S | S | fabG1 c.-15C>T | S | S | S | S | S |
|  |  |  |  |  |  |  |  |  |  |  |  |  |  |  |  |  |  |  |  |  |
| **MDR** | M1 | P | n/a | n/a | R | R | S | R | NT | NT | NT | NT | NT | R | NT | NT | NT | NT | NT | NT |
|  |  | O | 4.1.2.1 | H1 | rpoB p.Ser450Leu | katG p.Ser315Thr | embB p.Met306Ile | rpsL p.Lys43Arg | S | S | S | S | S | pncA p.Leu85Pro | S | S | S | S | S | S |
|  |  | I | 4.1.2.1 | H1 | rpoB p.Ser450Leu | katG p.Ser315Thr | embB p.Met306Ile | rpsL p.Lys43Arg | S | S | S | S | S | pncA p.Leu85Pro | S | S | S | S | S | S |
|  |  |  |  |  |  |  |  |  |  |  |  |  |  |  |  |  |  |  |  |  |
|  | M2 | P | n/a | n/a | R | R | S | R | NT | NT | NT | NT | NT | R | NT | NT | NT | NT | NT | NT |
|  |  | O | 2.2.1 | Beijing | rpoB p.Ser450Leu, rpoB p.Glu761Asp | katG p.Ser315Thr | embB p.Asp354Ala | S | S | S | S | S | eis c.-37G>T | S | ethA c.-7T>C | S | S | S | S | S |
|  |  | I | 2.2.1 | Beijing | rpoB p.Ser450Leu, rpoB p.Glu761Asp | katG p.Ser315Thr | embB p.Asp354Ala | S | S | S | S | S | eis c.-37G>T | S | ethA c.-7T>C | S | S | S | S | S |
|  |  |  |  |  |  |  |  |  |  |  |  |  |  |  |  |  |  |  |  |  |
|  | M3 | P | n/a | n/a | R | R | S | S | NT | NT | NT | NT | NT | R | NT | NT | NT | NT | NT | NT |
|  |  | O | 2.2.1 | Beijing | rpoB p.Ser450Leu, rpoB p.Glu761Asp | katG p.Ser315Thr | embB p.Asp354Ala | S | S | S | S | S | eis c.-37G>T | S | ethA c.-7T>C | S | S | S | S | S |
|  |  | I | 2.2.1 | Beijing | rpoB p.Ser450Leu, rpoB p.Glu761Asp | katG p.Ser315Thr | embB p.Asp354Ala | S | S | S | S | S | eis c.-37G>T | S | ethA c.-7T>C | S | S | S | S | S |
|  |  |  |  |  |  |  |  |  |  |  |  |  |  |  |  |  |  |  |  |  |
|  | M4 | P | n/a | n/a | R | R | S | R | NT | NT | NT | NT | NT | S | NT | NT | NT | NT | NT | NT |
|  |  | O | 4.2.1 | Ural-1 | rpoB p.Ser450Leu, rpoC p.Asp485Asn | inhA c.-154G>A, katG p.Ser315Thr | S | rpsL p.Lys88Arg | S | S | S | S | S | S | inhA c.-154G>A | S | S | S | S | S |
|  |  | I | 4.2.1 | Ural-1 | rpoB p.Ser450Leu, rpoC p.Asp485Asn | inhA c.-154G>A, katG p.Ser315Thr | S | rpsL p.Lys88Arg | S | S | S | S | S | S | inhA c.-154G>A | S | S | S | S | S |

P = phenotypic, O = Oxford Nanopore Technologies WGS, I = Illumina WGS, NT = Not tested, R = resistant, S = sensitive. Only resistance mutations of final confidence grading of ≥3 according to the WHO’s Catalogue of mutations in Mycobacterium tuberculosis complex and their association with drug resistance.
